# VarEPS: an evaluation and prewarning system of known and virtual variations of SARS-CoV-2 genomes

**DOI:** 10.1093/nar/gkab921

**Published:** 2021-10-11

**Authors:** Qinglan Sun, Chang Shu, Wenyu Shi, Yingfeng Luo, Guomei Fan, Jingyi Nie, Yuhai Bi, Qihui Wang, Jianxun Qi, Jian Lu, Yuanchun Zhou, Zhihong Shen, Zhen Meng, Xinjiao Zhang, Zhengfei Yu, Shenghan Gao, Linhuan Wu, Juncai Ma, Songnian Hu

**Affiliations:** Microbial Resource and Big Data Center, Institute of Microbiology, Chinese Academy of Sciences, Beijing 100101, China; Chinese National Microbiology Data Center (NMDC), Beijing 100101, China; Microbial Resource and Big Data Center, Institute of Microbiology, Chinese Academy of Sciences, Beijing 100101, China; State Key Laboratory of Microbial Resources, Institute of Microbiology, Chinese Academy of Sciences, Beijing 100101, China; Microbial Resource and Big Data Center, Institute of Microbiology, Chinese Academy of Sciences, Beijing 100101, China; Chinese National Microbiology Data Center (NMDC), Beijing 100101, China; Microbial Resource and Big Data Center, Institute of Microbiology, Chinese Academy of Sciences, Beijing 100101, China; State Key Laboratory of Microbial Resources, Institute of Microbiology, Chinese Academy of Sciences, Beijing 100101, China; University of Chinese Academy of Sciences, Beijing 100049, China; Microbial Resource and Big Data Center, Institute of Microbiology, Chinese Academy of Sciences, Beijing 100101, China; Chinese National Microbiology Data Center (NMDC), Beijing 100101, China; Microbial Resource and Big Data Center, Institute of Microbiology, Chinese Academy of Sciences, Beijing 100101, China; State Key Laboratory of Microbial Resources, Institute of Microbiology, Chinese Academy of Sciences, Beijing 100101, China; University of Chinese Academy of Sciences, Beijing 100049, China; CAS Key Laboratory of Pathogenic Microbiology and Immunology, Institute of Microbiology, Chinese Academy of Sciences, Beijing 100101, China; CAS Key Laboratory of Pathogenic Microbiology and Immunology, Institute of Microbiology, Chinese Academy of Sciences, Beijing 100101, China; CAS Key Laboratory of Pathogenic Microbiology and Immunology, Institute of Microbiology, Chinese Academy of Sciences, Beijing 100101, China; State Key Laboratory of Protein and Plant Gene Research, Center for Bioinformatics, School of Life Sciences, Peking University, Beijing 100871, China; Computer Network Information Center, Chinese Academy of Sciences, Beijing 100190, China; Computer Network Information Center, Chinese Academy of Sciences, Beijing 100190, China; Computer Network Information Center, Chinese Academy of Sciences, Beijing 100190, China; Microbial Resource and Big Data Center, Institute of Microbiology, Chinese Academy of Sciences, Beijing 100101, China; Chinese National Microbiology Data Center (NMDC), Beijing 100101, China; Microbial Resource and Big Data Center, Institute of Microbiology, Chinese Academy of Sciences, Beijing 100101, China; Chinese National Microbiology Data Center (NMDC), Beijing 100101, China; Microbial Resource and Big Data Center, Institute of Microbiology, Chinese Academy of Sciences, Beijing 100101, China; State Key Laboratory of Microbial Resources, Institute of Microbiology, Chinese Academy of Sciences, Beijing 100101, China; Microbial Resource and Big Data Center, Institute of Microbiology, Chinese Academy of Sciences, Beijing 100101, China; Chinese National Microbiology Data Center (NMDC), Beijing 100101, China; Microbial Resource and Big Data Center, Institute of Microbiology, Chinese Academy of Sciences, Beijing 100101, China; State Key Laboratory of Microbial Resources, Institute of Microbiology, Chinese Academy of Sciences, Beijing 100101, China; Chinese National Microbiology Data Center (NMDC), Beijing 100101, China; Microbial Resource and Big Data Center, Institute of Microbiology, Chinese Academy of Sciences, Beijing 100101, China; State Key Laboratory of Microbial Resources, Institute of Microbiology, Chinese Academy of Sciences, Beijing 100101, China; University of Chinese Academy of Sciences, Beijing 100049, China

## Abstract

The genomic variations of SARS-CoV-2 continue to emerge and spread worldwide. Some mutant strains show increased transmissibility and virulence, which may cause reduced protection provided by vaccines. Thus, it is necessary to continuously monitor and analyze the genomic variations of SARS-COV-2 genomes. We established an evaluation and prewarning system, SARS-CoV-2 variations evaluation and prewarning system (VarEPS), including known and virtual mutations of SARS-CoV-2 genomes to achieve rapid evaluation of the risks posed by mutant strains. From the perspective of genomics and structural biology, the database comprehensively analyzes the effects of known variations and virtual variations on physicochemical properties, translation efficiency, secondary structure, and binding capacity of ACE2 and neutralizing antibodies. An AI-based algorithm was used to verify the effectiveness of these genomics and structural biology characteristic quantities for risk prediction. This classifier could be further used to group viral strains by their transmissibility and affinity to neutralizing antibodies. This unique resource makes it possible to quickly evaluate the variation risks of key sites, and guide the research and development of vaccines and drugs. The database is freely accessible at www.nmdc.cn/ncovn.

## INTRODUCTION

As an RNA virus, severe acute respiratory syndrome coronavirus 2 (SARS-CoV-2) has a relatively high mutation rate (1) with a mean annual average evolutionary rate of 1 × 10^–3^ substitutions per base per year under conditions of neutral genetic drift (2). Since the initial outbreak in December 2019, a substantial number of SARS-CoV-2 variants have emerged. As of August 2021, a total of 2 635 714 SARS-CoV-2 genome sequences have become available in the Global Initiative of Sharing All Influenza Data (GISAID database), and 29 212 mutations have accumulated over the past year and a half. However, most mutations in SARS-CoV-2 occur at a very low frequency and cause no significant effect on the virus (3). Only a small number of mutations, especially those in the spike (S) protein, can change the infectivity of the virus and hence increase transmission or reduce the binding affinity of the S protein receptor-binding domain (S-RBD) for neutralizing antibodies. For instance, a point mutation in the S protein, D614G, shifts the conformation of the S protein toward an angiotensin-converting enzyme 2 (ACE2)-binding fusion-competent state and hence enhances SARS-CoV-2 infectivity in human lung cells (4). Research using computational simulation has suggested that some mutations, including Q24T, T27D/K/W, D30E, H34S7T/K, E35D, Q42K, L79I/W, R357K and R393K in ACE2, and L455D/W, F456K/W, Q493K, N501T and Y505W in S-RBD, increase the binding affinity between ACE2 and S-RBD. Experimental evidence has shown that these *in silico* simulations are highly accurate (5).

Efficient and accurate diagnosis of COVID-19 is crucial for controlling the pandemic in early time. Reverse transcription polymerase chain reaction (RT-PCR) technology is the most widely used among the common diagnostic methods (6). Mutations at the probe or primer sites could have effects on the accuracy of diagnosis, such as loss of primer efficacy. As a result, continued surveillance of genomic mutation is crucial for disease control and vaccine and drug studies.

We here established the variations evaluation and prewarning system (VarEPS) and conducted a comprehensive analysis of the effects of variants on physicochemical properties, translation efficiency, secondary structure, difficulty in developing variations, binding capacity of ACE2 and binding capacity of neutralizing antibodies. To our knowledge, this is the most comprehensive analysis and risk evaluation of SARS-CoV-2 genome variants. Instead of the classical risk evaluation by variation frequency, we followed a new perspective on the effect of mutations on protein structure and function. Moreover, we constructed two random forest classifiers to verify the effectiveness of these characteristic quantities for accurate risk evaluation. This AI-based classifier can be used to accurately group strains by their transmissibility and affinity to neutralizing antibodies.

More importantly, we analyzed not only known variants but also virtual variants; as a result, by closely observing newly submitted genome sequences, we can identify emerging dangerous variants at an early stage. This platform can also yield vital information for virologists using pseudoviruses to test vaccines and drugs. Currently, VarEPS is the only database which provides these unique resources on virtual variants and is thus expected to be of great interest for virologists, especially those involved in vaccine and drug development.

## DATABASE INTERFACE AND FEATURES

### Database interface

**Figure 1.**
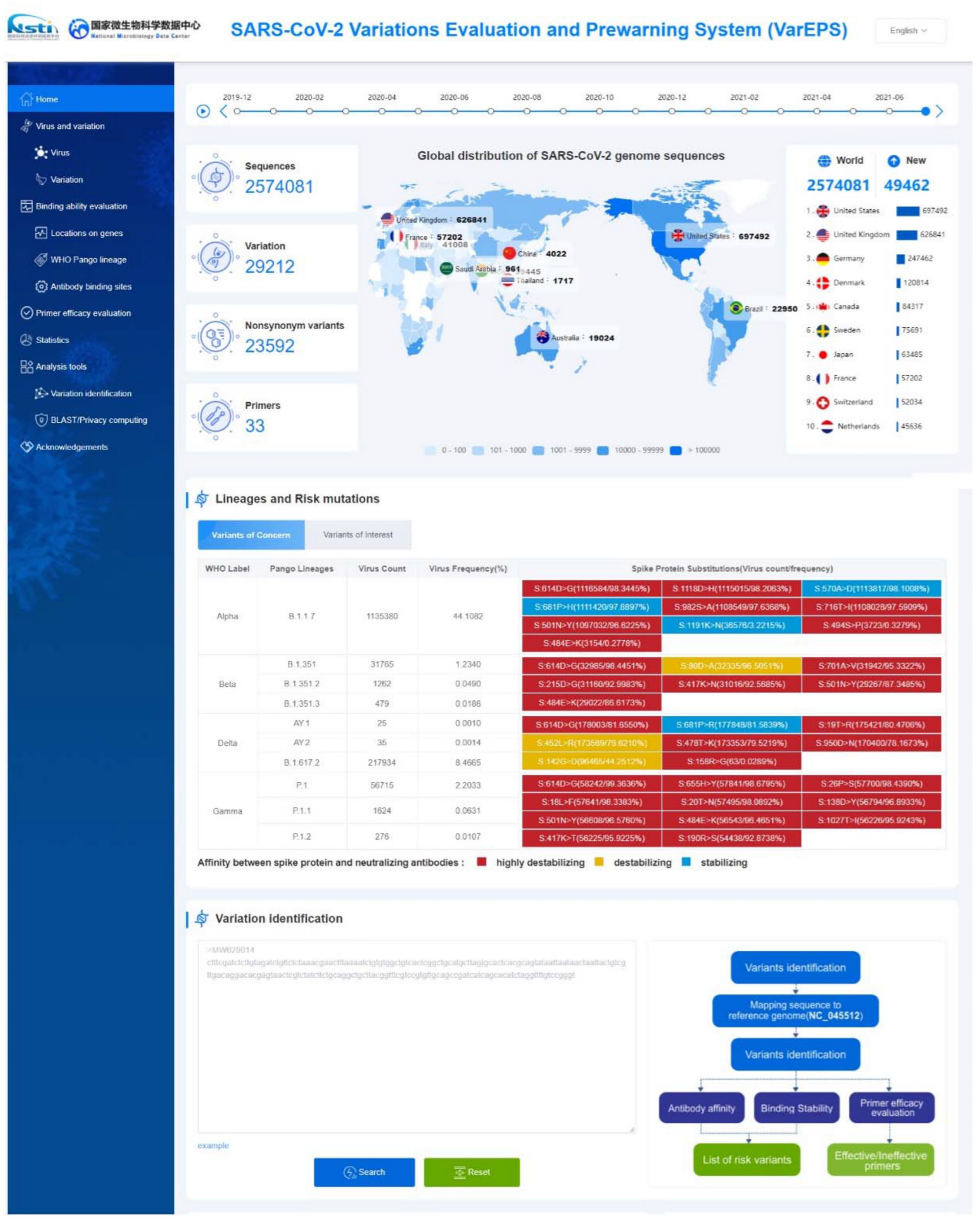
Features of the variations evaluation and prewarning system (VarEPS) portal. We show a global distribution of genome sequences by time frame and geography. The risk level and frequency of characteristic variants of each lineage are listed. Users can submit a sequence for variation analysis directly on the homepage.

The web interface of VarEPS is composed of five main sections: ‘Virus and variation’, ‘Binding ability evaluation’, ‘Primer efficacy evaluation’, ‘Statistics’ and ‘Analysis tools’ (Figure 1). The ‘Virus and variation’ section starts with search interfaces for metadata attributes of viral sequences and nucleotide variants. The resulting viral sequences with associated metadata are displayed as a table, including lineage, single nucleotide polymorphisms (SNP) number, and variation information for both nucleotides and amino acids. Each viral sequence is linked to an individual page containing all of the related mutations and primer evaluation results. A machine learning model is used to give an overall risk level prediction for each virus. The query on nucleotide variation returns a variant list with metadata related to the number of variations and the associated amino acid mutations. Each variation is linked to a page containing graphs of distribution over time and by country, and listing related viral sequences.

The ‘Binding ability evaluation’ section assesses the risk level of each virus variant. Variants may be queried and browsed by their location on genes, lineages and antibody binding sites. After query by different metadata, a list containing all amino acid mutations is returned. Antibody affinity, binding stability with ACE2, risk of amino acid substitution, and the first-seen and last-seen time are calculated and displayed. Each amino acid variation is linked to a page containing details of these values or the risk level.

The ‘Primer efficacy evaluation’ section assesses how mutations affect primer design for RT-PCR. Primer information is obtained from the USA Centers for Disease Control and Prevention (CDC), the Chinese Center for Disease Control and Prevention (CDC China), the World Health Organization (WHO) and others. If mutations are present in the 5′- and 3′- end, the primers might be of low specificity or lose efficacy entirely.

### Online data analysis pipelines

Online analysis tools are provided for users to submit sequences for variation analysis. Sequences are aligned against the reference genome (NC_045512.2) using NUCmer from the MUMmer package (7). Thereafter, a catalog of all SNPs and indels internal to the reference genome is generated. The system evaluates variants and generates risk level results by assessing amino acid substitution, binding affinity for ACE2 and secondary structure change. For variants of the S-RBD, the affinity with 15 neutralizing antibodies under development is calculated. Nucleotide mismatches with primers or probes are reported to warn of possible false negative results in diagnostic detection of SARS-CoV-2 by real-time RT-PCR. An evaluation report of the submitted virus is sent to users via e-mail after all analyses are complete.

### Statistics

A statistics page organized by ‘Lineage’, ‘Variations’ and ‘Primer’ provides an overview of statistical analysis of variants. The ‘Lineage’ page displays the distribution of different lineages by country and through time. The ‘Variations’ page gives a set of graphs on variant distribution and risk level of different lineages. The ‘Primer’ page lists primer evaluation results of different lineages. Interactive interfaces are provided to allow the user to further explore the features of various groups.

## DATA CONTENT AND ANALYSIS

**Figure 2.**
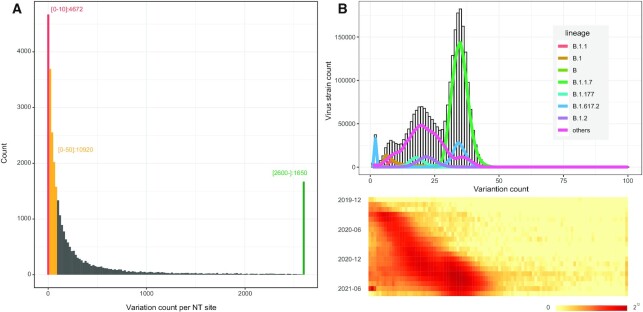
Statistics of nucleotide mutation numbers in SARS-CoV-2 genomes. (**A**) Histogram of the mutation count at all nucleotide positions. Red, orange and green bars refer to the frequency of mutation count below 10, below 50 and above 2600, respectively. (**B**) Histogram of total mutation count in one strain. The heatmap shows the distribution of total mutation count in each month. Mutation counts are accumulating over time and coordinate with lineages.

We calculate the occurrence of each mutation site in nucleotide/amino acid variants against the reference sequence. Currently, there are 29 212 variants observed on nearly 30 000 nucleotides of the SARS-CoV-2 whole length genome sequence. However, many variants are of a very low frequency. Among the 29 212 nucleotide variants, 4672 (16.0%) sites occur <10 times and 10 920 (37.4%) sites occur <50 times. Only 1650 (5.7%) sites occur >2600 times (with a frequency of 0.1%) and 33 (0.1%) sites occur >24 000 times (with a frequency of 1%) (Figure 2A). The SARS-CoV-2 mutation rate is vital to determining how quickly the transmissibility of a virus changes and immune evasion occurs. The mean annual mutation rate is reported to be 1 × 10^–3^ substitutions per base per year (2), and apparently, the observed mean mutation occurrence rate is consistent with the estimated rates and is closely associated with lineage (Figure 2B).

Among amino acid variants, the most frequent variant is D614G. The next most frequent, N501Y, is located in the S-RBD, whereas the frequency of all other S-RBD variants is <10% (Table 1). Still, a large number of high frequency mutations are located outside of the S protein. Variants that appear in viral populations with a high frequency or that are located in domains with critical effects on viral structure or function should be given our utmost attention.

**Table 1.** Variants of SARS-CoV-2 genome and most common variants located on S-RBD.

	Whole genome high frequency variants			RBD high frequency variants		
NO	Variants	Counts	Frequency	Variants	Counts	Frequency
**1**	S:D614G	2467291	95.85%	S:N501Y	1200001	46.62%
**2**	ORF1ab:P314L	2437459	94.69%	S:L452R	269897	10.49%
**3**	N:R203K	1434386	55.72%	S:T478K	206979	8.04%
**4**	N:G204R	1432232	55.64%	S:E484K	151017	5.87%
**5**	S:N501Y	1200001	46.62%	S:S477N	68895	2.68%
**6**	S:P681H	1178130	45.77%	S:K417T	57507	2.23%
**7**	ORF1ab:T1001I	1125623	43.73%	S:K417N	33585	1.30%
**8**	S:D1118H	1124839	43.70%	S:N439K	33447	1.30%
**9**	S:A570D	1122643	43.61%	S:S494P	12880	0.50%
**10**	S:T716I	1122555	43.61%	S:F490S	7757	0.30%
**11**	ORF8:Y73C	1120251	43.52%	S:E484Q	7179	0.28%
**12**	ORF1ab:A1708D	1118801	43.46%	S:A520S	5443	0.21%
**13**	N:S235F	1118673	43.46%	S:N440K	4610	0.18%
**14**	S:S982A	1116061	43.36%	S:A522S	4436	0.17%
**15**	ORF8:R52I	1113847	43.27%	S:N501T	4194	0.16%
**16**	N:D3L	1112519	43.22%	S:L452Q	3704	0.14%
**17**	ORF1ab:I2230T	1099897	42.73%	S:V367F	2499	0.10%
**18**	ORF3a:Q57H	456450	17.73%	S:R346K	2357	0.09%
**19**	ORF1ab:E265I	365975	14.22%	S:P384L	2253	0.09%
**20**	S:L452R	269897	10.49%	S:R346S	2188	0.09%

**Figure 3.**
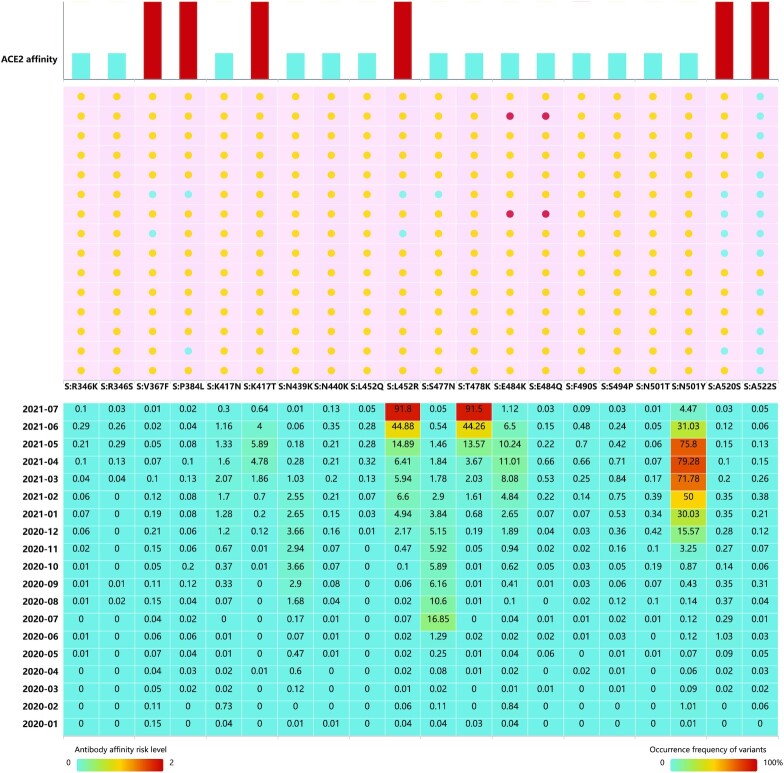
Binding stability to ACE2 and antibody affinity risk level for key mutations on S-RBD. Risk levels of reduced antibody affinity for 15 antibodies were calculated. The risk levels of antibody affinity and increased binding stability to ACE2 are ranked 0 to 2. Frequency of these variants over time are provided.

SARS-CoV-2 S-RBD is the molecular target for most SARS-CoV-2 vaccines and antibodies currently in use or under development. We compared key amino acid mutations (the top 20 most frequent variants) in the S-RBD for their effects on S protein affinity with neutralizing antibodies and ACE2 (Figure 3). The simulated results showed that the most frequent variants reduced the binding affinity of the S protein for neutralizing antibodies. This result should be followed up with *in vivo* experiments to test the simulation results and examine the effects. Other variants (e.g. L452R and K417T) exhibited increased affinity with ACE2, indicating enhanced infectivity of these variants. Combined with the distributions with time span, it is critical to pay close attention to the risk presented by emerging variants that rapidly increase in frequency.

**Figure 4.**
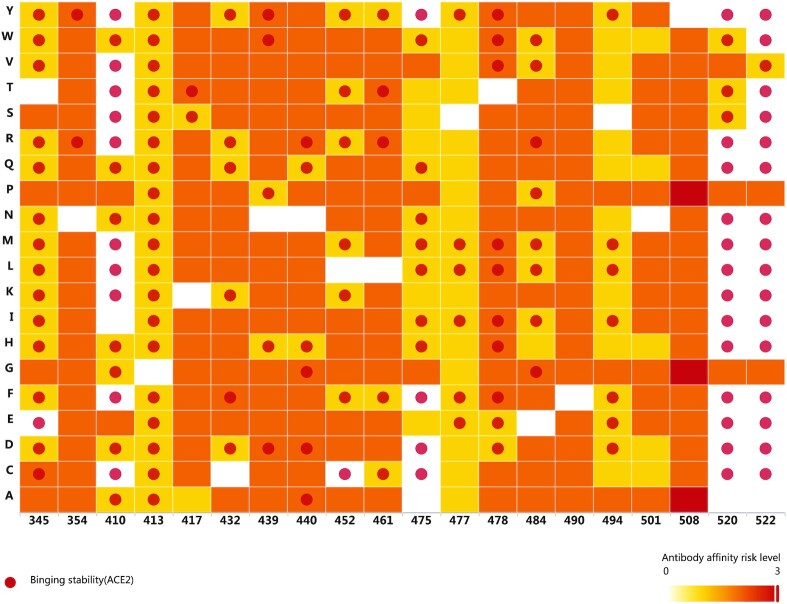
Binding stability to ACE2 and antibody affinity risk level for key known mutations and virtual mutations on S-RBD. A red dot indicates is increased binding stability to ACE2. Overall risk levels of reduced antibody affinity for 15 antibodies are ranked 0 to 3. Both known and virtual mutations were evaluated.

Apart from the existing mutations, this platform allows evaluation of new mutations as they appear in the future. Evaluating the risk level of virtual mutations could facilitate drug and/or vaccine development. From the simulation results (Figure 4), we estimated that antibody affinity will be reduced as a result of most of these virtual mutations. Binding stability to ACE2 will also be affected by mutations in some key positions (e.g. 345, 413, 520 and 522).

L452R is an important mutation that has commonly appeared in the recently prevalent Alpha, Delta, Epsilon, Iota and Kappa strains, and it is reported that the variants can reduce sensitivity to neutralizing antibodies (8). This mutation may increase affinity for ACE2 receptors and accordingly increase infectivity (9). Consistent with these experimental results, our prewarning system results indicated that the variation may be associated with increased infectivity and decreased affinity with some neutralizing antibodies. Additionally, we predicted all possible variants at this site; the data revealed that the risk level for some variants was even higher than the currently widespread L452R, including L452Q, which is one of the characteristic variants of Lambda strains. Others were virtual variants that have not yet, such as L452A, L452N and L452D. Emerging variants should be closely monitored for such mutations with high predicted risk levels.

**Figure 5.**
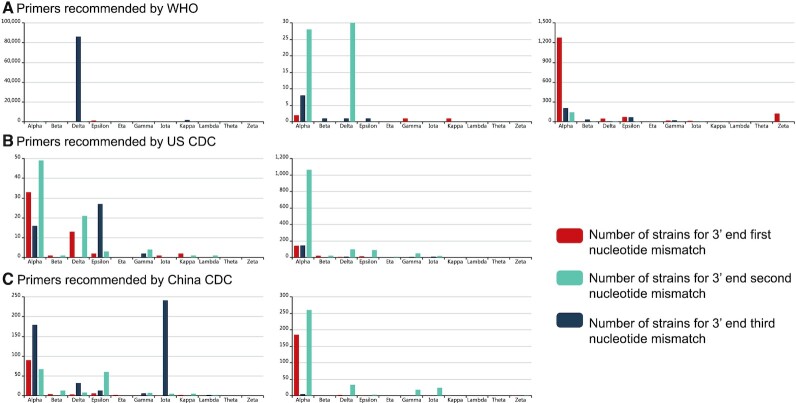
Nucleotide mismatch statistics for primers. Nucleotide mismatches were compared for the 3′ end of primers. The number of strains for each lineage were calculated.

Finally, we list variants that could affect the performance of the primers recommended by WHO, CDC and CDC China. These data are organized by number of mismatched nucleotides for different lineages (Figure 5). Most of these mismatches occur at the first nucleotide of the 3′ end for Alpha strains. However, the number of affected viruses is very low. Considering the high percentage of SNPs of the SARS-CoV-2 genome, it is not practical to avoid all SNPs on every primer/probe binding site. Although false negative results may occur, many molecular tests tolerate a few single nucleotide mismatches, which have low or even no impact at all on their performance.

## METHODS

### Data sources and data processing

**Figure 6.**
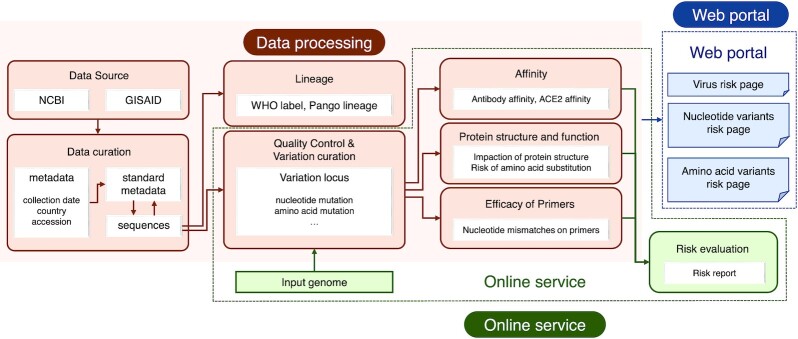
Schematic representation of VarEPS for data processing and online analysis service. SARS-CoV-2 genome sequences were integrated to perform metadata curation and quality control procedures. Sequence data were mapped to the reference genome for variation annotation. Each annotated variant was used to calculate effects on translation efficiency, secondary structure, binding capacity of ACE2 and neutralizing antibodies and efficacy of primers. Our web portal provides multiple query selections to display results on both known and virtual mutations. The system also provides online analysis service for custom submitted sequences.

We extracted 2 635 714 SARS-CoV-2 sequences from the EpiCov™ section of the GISAID portal (10), and 956 676 SARS-CoV-2 sequences from the US National Center for Biotechnology Information (11) and NMDC (www.nmdc.cn). After low quality and duplicated sequences were removed, the final filtered raw sequence data set comprised 2 574 081 sequences. Each sequence was mapped against the reference genome from Wuhan, China (NCBI accession No. NC_045512.2) to identify mutations and deletions in the SARS-CoV-2 genome. We used the same site-numbering scheme as the reference genome to generate the lists of nucleotide variants and amino acids variants. Each mutation was then examined according to the following aspects (Figure 6):

**Changes in free energy of binding with neutralizing antibodies caused by single amino acid mutation:** Saambe-3D (12) was used to predict changes in free energy of binding caused by single amino acid mutation and disruption of protein–protein interaction (PPI). Mutation types included destabilizing mutation (ΔΔ*G* > 0), stable mutation (−1.5 < ΔΔ*G* < 0), highly destabilizing mutation (ΔΔ*G* > 1.5) and highly stable mutation (ΔΔ*G* < −1.5). Subsequently, we predicted the affinity with 15 neutralizing antibodies (13–27), some of which have been approved as therapeutic antibodies for COVID-19 (casirivimab [28], imdevimab [28], bamlanivimab [29], etesevimab [29] and sotrovimab [30]). Finally, we assigned an overall ranked risk level from 1 to 3 based on the average ΔΔ*G* values for all 15 antibodies.
**Changes in free energy of binding with S protein and ACE2 induced by single amino acid mutation:** Saambe-3D was utilized to predict changes in free energy of binding caused by single amino acid mutation and whether that mutation could disrupt the PPI. Mutation types included destabilizing mutation (ΔΔ*G* > 0), stable mutation (−1.5 < ΔΔ*G* < 0), highly destabilizing mutation (ΔΔ*G* > 1.5) and highly stable mutation (ΔΔ*G* < −1.5). We assigned risk level 2 to highly stable mutations (ΔΔ*G* < −1.5) and risk level 1 to stable mutation (−1.5 < ΔΔ*G* < 0).
**Difficulty of occurrence of nucleotide diversity:** This was represented by a ‘nonsynonymous density’ value reflecting the difficulty of the occurrence of nucleotide diversity and was evaluated by calculating the density of synonymous mutations and missense mutations under a sliding window. High-frequency variants that occurred before July 2021 were used as major alleles for statistical analysis. The density reflects the difficulty of occurrence of a mutation in a certain segment. High frequency densities indicate rapidly accumulating mutations in the region and low frequency densities may indicate a SNP desert (31), i.e. regions where potential selection of elimination occurs, implying that the virus has long-term and stable adaptive changes in this region.
**Risk of replacement of amino acid:** PAM (32) and BLOSUM (33) matrices were employed to evaluate the risk of amino acid replacement. If replacement of two amino acids frequently occurred, it indicated that such amino acid replacements are stable. The replacement was assigned a low risk level and vice versa.
**Effects of mutations on biological function of proteins:** ‘Impact on protein function’ was calculated using PROVEAN (34) to predict the effects of amino acid variants on the biological functions of proteins. The threshold for destructiveness and neutrality was set at −2.5.
**Effect of variation on secondary structure:** Bepipred2.0 (35) was used for the ‘secondary structure prediction’ of the mutated protein and for comparison with the published X-ray diffraction data for the protein.
**Effects of variation on potential continuous and discontinuous epitopes:** ElliPro (36) was used to predict ‘changes of antigen continuous epitopes’ and ‘changes of antigen discontinuous epitopes’ before and after the variation occurred.
**Effect of variation on effectiveness of detection reagents:** For PCR ‘Primer efficacy evaluation’, the location and the frequency of the variant were considered comprehensively. If the variant occurred in the last three bases of the 3′ end, an early warning score will be given. In addition, the number of mutations was also assessed, and the corresponding score was given based on the number of variants at the last three bases in the 3′ end. The warning rating for RT-PCR primers was based on these scores.

### Machine learning model for risk evaluation

We performed a comprehensive analysis of viral strain risk level by evaluating the difficulty of occurrence of nucleotide variants, possibility of amino acid replacement, change in protein secondary structure, and changes in ACE2 and neutralizing antibody free energy of binding caused by individual amino acid mutations. Each strain was given a series of characteristic quantities according to every mutation it carries. We constructed two random forest classifiers to verify the effectiveness of these characteristic quantities and used these parameters to group strains by their transmissibility and affinity with neutralizing antibodies.

The strains belong to eight WHO VOI/VOC were grouped into six groups according to two grouping modes: the normal transmissibility group, the mildly increased transmission group, the severely increased transmission group, the normal affinity group, the mildly decreased affinity group and the severely decreased affinity group. Up to 50 000 complete genomic sequences were randomly extracted from the GISAID database for each of the eight VOI/VOC strains, and approximately 200 000 sequences were used to construct the model. All variant sites in the whole genome sequence of a strain were identified and parameters including the difficulty of occurrence of nucleotide variants, the possibility of amino acid replacement, the effect of variants on protein secondary structure, and changes in ACE2 and neutralizing antibody binding free energy caused by individual amino acid mutations were calculated for each variant site, which were then used to assign values to a strain sequence and construct the dataset. The Boruta algorithm was used to filter the feature measurements and the random forest algorithm was used to construct the classification model. To assess the reliability and stability of the model, 1000 random iterations were performed (70% were randomly selected as the training set and the remaining 30% as the testing set in each iteration). The prediction performance of the model was measured by area under the curve, accuracy, precision and sensitivity. Details of Machine Learning Model were provided in supplementary material (Supplementary Tables S1–S4 and Figures S1–S3).

## CONCLUSION AND FUTURE DIRECTIONS

As of 5 August 2021, the number of confirmed COVID-19 patients worldwide reached 200 million with >4 million deaths. Over 70 vaccines are currently under development and 4 billion vaccine doses have already been administered (https://coronavirus.jhu.edu/map.html). Rapid diagnosis and vaccination are still the most effective methods for controlling the pandemic. As a result, it remains crucial to understand whether SARS-CoV-2 variants impact the affinity of current neutralizing antibodies under development or the performance of current diagnostic methods. It is also critical to pay close attention to variants that may escape from protective immune responses induced by population-level immunity. The VarEPS system presented here allows close monitoring and evaluation of the current global status of genetic variations of SARS-CoV-2.

VarEPS enables the user to focus on the updated global status of SARS-CoV-2 genome sequences and variation analysis. It provides different levels of variant evaluation for translation efficiency, secondary structure, binding capacity of ACE2, binding capacity of neutralizing antibodies and efficacy of RT-PCR primers. Combined with the online analysis tools, the system can serve as both a navigation and recommendation tool for global virus variant surveillance. Moreover, the system can aid in designing robust vaccines and neutralizing monoclonal antibodies in the future. Based on the risk level evaluation of virtual variants, it provides key information for the design of prophylactic antibodies and vaccines that target variations with higher risk levels.

We will continuously update the system with new data on various resources of SARS-CoV-2 genome sequences. The machine learning model presented here is the first to successfully evaluate binding affinity and to group strains based on this attribute. The model will be further developed for broader evaluations. As more *in vitro* and *in vivo* studies are conducted, the *in silico* models will be iteratively optimized, and the simulation and prediction features will improve in accuracy with solid support from experimental results.

## DATA AVAILABILITY

There are no access restrictions for academic use of the platform. Access to VarEPS is free at www.nmdc.cn/ncovn.

## Supplementary Material

gkab921_Supplemental_File
