# Supplementary material for: VarEPS: an evaluation and prewarning system of known and virtual variations of SARS-CoV-2 genomes
Source: Nucleic Acids Res. 2021 Oct 11;50(D1):D888–97. doi: 10.1093/nar/gkab921 (PMC8728250; doi:10.1093/nar/gkab921)
Supplement: gkab921_Supplemental_File [file gkab921_supplemental_file.docx]

**Supplementary for Machine Learning Model for Risk Evaluation**

Steps of establishing the classifier (Figure S1):

1. Data collection and grouping: Based on previous studies^[1]^ and Eric Topol’s opinion(<https://twitter.com/EricTopol>), strains belong to the eight WHO VOI/VOC were divided into six groups according to two grouping models: the normal transmissibility group, the mildly increased transmissibility group, the severely increased transmissibility group, the normal affinity group, the mildly decreased affinity group and the severely decreased affinity group.(Table S1)

Table S1. The six groups of eight WHO VOI/VOC according to two grouping models

| Affinity to neutralizing antibodies | Normal | Alpha(B.1.1.7); Iota(B.1.526) |
| --- | --- | --- |
|  | Mild decrease | Gamma(p.1); Delta(B.1.617.2); Eta(B.1.525);  Kappa(B.1.617.1); Lambda(C.37) |
|  | Severe decrease | Beta(B.1.351) |
| Transmissibility | Normal | Beta(B.1.351); Iota(B.1.526) |
|  | Mild Increased | Gamma(p.1); Eta(B.1.525); Kappa(B.1.617.1); Lambda(C.37) |
|  | Severe Increased | Alpha(B.1.1.7); Delta(B.1.617.2) |

Up to 50,000 complete SARS-CoV-2 genomic sequences were randomly extracted from the GISAID database for each of the eight VOI/VOC strains, and approximately 200,000 sequences were used to construct the model.

1. Variation identification and annotation: Each sequence was mapped against the reference genome from Wuhan, China (NCBI accession No. NC_045512.2) to identify mutations in the SARS-CoV-2 genome. We used the same site-numbering scheme as the reference genome to generate the lists of nucleotide variants and amino acids variants.
2. Parameter calculation and feature matrix construction: All variant sites in the whole genome sequence of a strain were identified and parameters including the difficulty of occurrence of nucleotide variants, the possibility of amino acid replacement, the effect of variants on protein secondary structure, and changes in ACE2 and neutralizing antibody binding free energy caused by individual amino acid mutations were calculated for each variant site, which were then used to assign values to a strain sequence and construct the dataset. (The specific algorithm and software for the parameters above can be found in Method)
3. Feature selection: We removed features with high correlation (R^2^>0.9, P<0.05), then we performed feature selection using Boruta. Finally, the classifier for affinity to neutralizing antibodies used 9 features, the classifier for transmissibility used 8 features. (Table S2)

Table S2. The features had been used for the classifiers

| Groups | Selected features |
| --- | --- |
| Transmissibility | ΔΔG_6LZG、ΔΔG_6YLA、ΔΔG_7K8M、ΔΔG_7l3n、ΔΔG_7NEH、ΔΔG_7NXC、BLOSUM62、provean_holistic |
| Affinity to neutralizing antibodies | ΔΔG_6LZG、ΔΔG_6YLA、ΔΔG_7K8M、ΔΔG_7l3n、ΔΔG_7NEH、ΔΔG_7NXC、ΔΔG_7bv2、BLOSUM62、provean_holistic |

ΔΔG_*：Changes in Free Energy Binding of Neutralizing Antibodies; BLOSUM62：BLOSUM matrix

provean_holistic：results of PROVEAN software.

1. Classifier construction: Most of the features we used are dispersedly distributed with limited values rather than continuous variables, which makes the Random Forest model a more suitable option compared to the Neural Network model. The comparison we had made between Support Vector Machine model and Random Forest model shows that, the Radom Forest model provides Lower error rate (Minimum error rate in 100 cycles: error_rate _SVM_ transmissibility =0.1438; error_rate_SVM_neutralizing_antibodies_affinity = 0.1276; error_rate_RF_ transmissibility =0.0787; error_rate _RF_ neutralizing_antibodies_affinity = 0.0650). Thus, we determined to construct Radom Forest model as the classifier model. To assess the reliability and stability of the model, 1000 random iterations were performed (70% were randomly selected as the training set and the remaining 30% as the testing set in each iteration). The prediction performance of the model was measured by area under the curve, accuracy, precision, and sensitivity. (Figure S2 and Figure S3, Table S3 and Table S4)


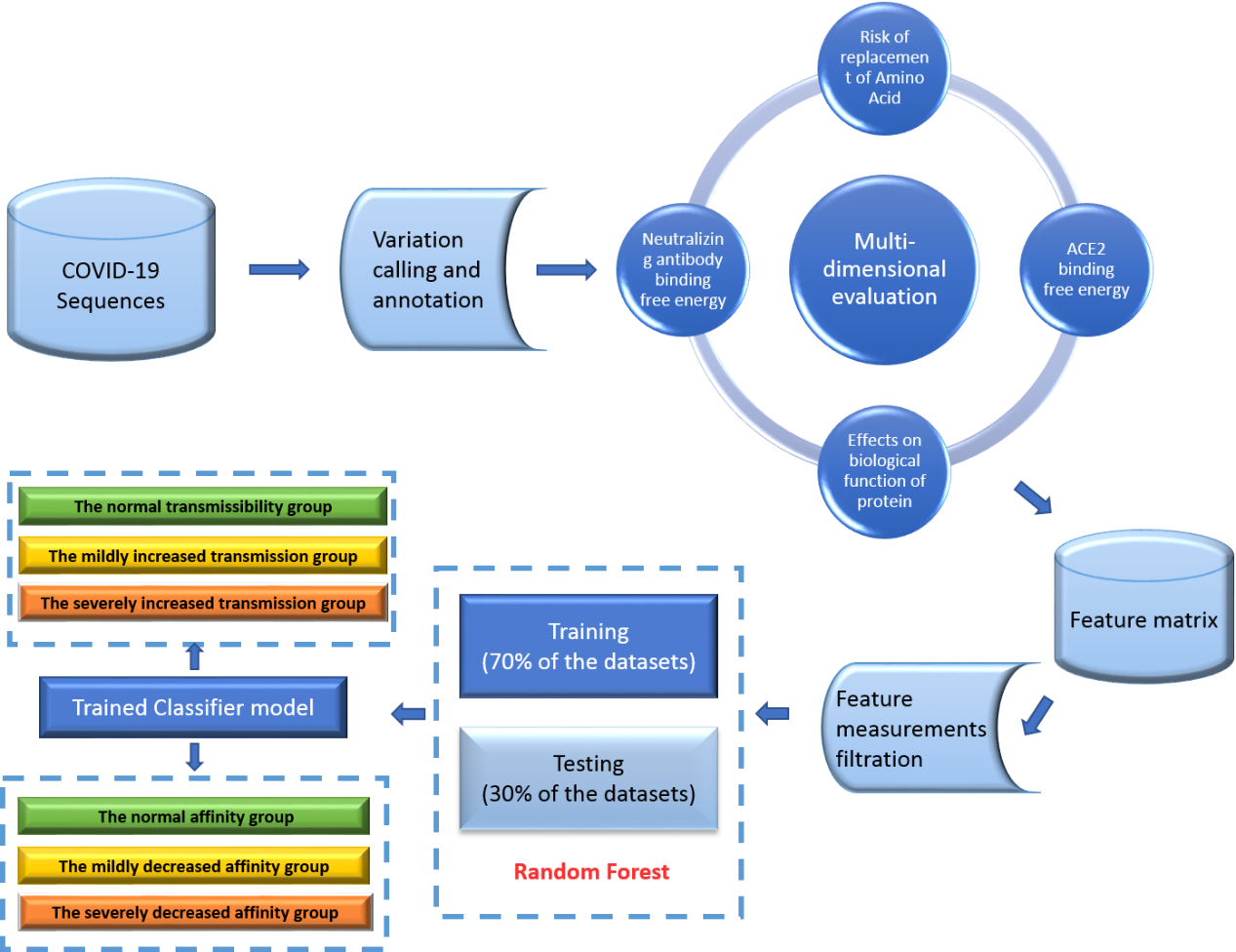


Figure S1: Graphical abstract of the AI-based classifier’s for SARS-Cov-2 variants


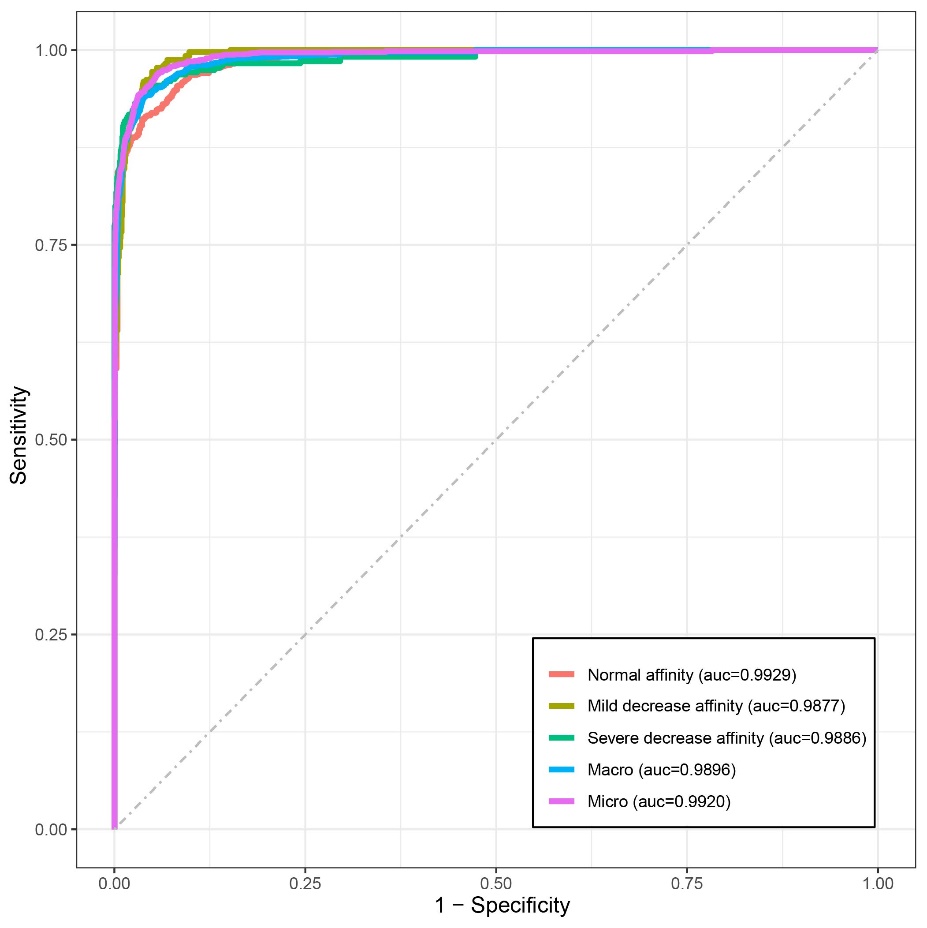


Figure S2: ROC curves for affinity to neutralizing antibodies grouping model


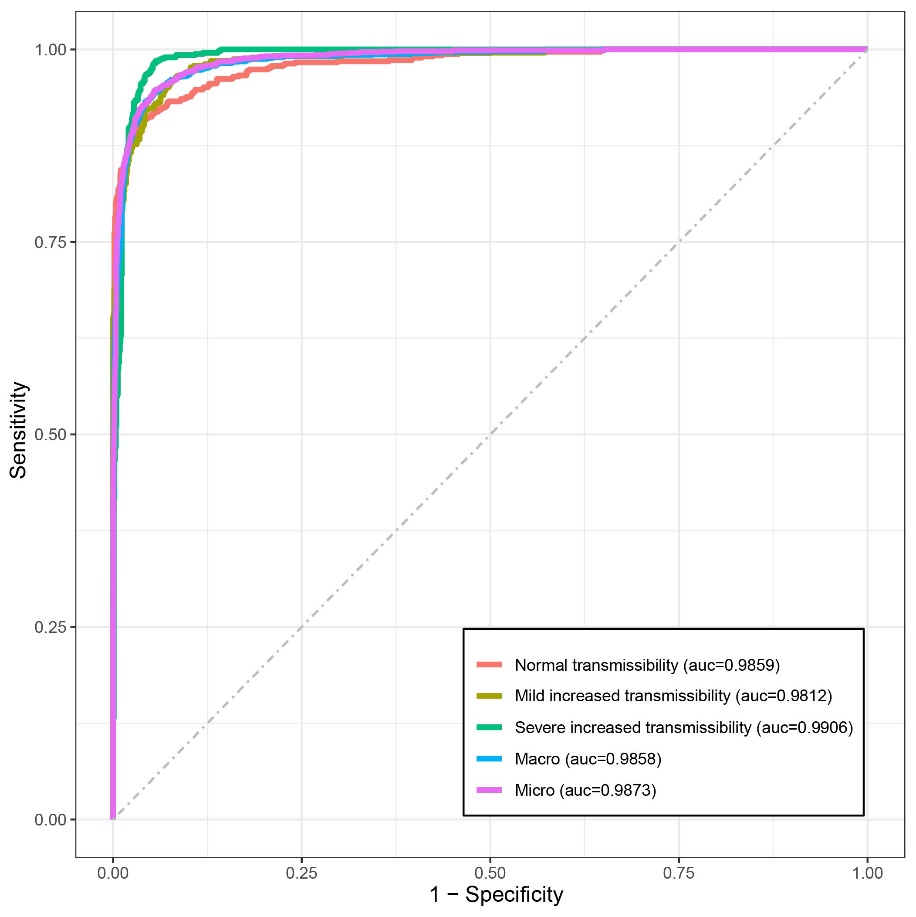


Figure S3: ROC curves for transmissibility grouping model

Table S3. Assessment of affinity to neutralizing antibodies grouping model

| Affinity to neutralizing antibodies grouping model | AUC | Sensitivity | Specificity | Precision | F1 | Accuracy |
| --- | --- | --- | --- | --- | --- | --- |
| Mild decrease | 0.9877 | 0.9640 | 0.9032 | 0.9436 | 0.9537 | 0.9413 |
| Normal | 0.9929 | 0.8858 | 0.9818 | 0.9233 | 0.9041 | 0.9629 |
| Severe decrease | 0.9886 | 0.9057 | 0.9866 | 0.9351 | 0.9202 | 0.9724 |

Table S4. Assessment of transmissibility grouping model

| Transmissibility grouping model | AUC | Sensitivity | Specificity | Precision | F1 | Accuracy |
| --- | --- | --- | --- | --- | --- | --- |
| Mild increase | 0.9812 | 0.9080 | 0.9636 | 0.9338 | 0.9207 | 0.9435 |
| Normal | 0.9859 | 0.8726 | 0.9745 | 0.9236 | 0.8974 | 0.9480 |
| Severe increase | 0.9906 | 0.9795 | 0.9484 | 0.9202 | 0.9490 | 0.9601 |

[1] D Corti，LA Purcell，G Snell，D Veesler. (2021) Tackling COVID-19 with neutralizing monoclonal antibodies. *Cell*, **184**, 12
